# Inflammasome Deletion Promotes Anti-tumor NK Cell Function in an IL-1/IL-18 Independent Way in Murine Invasive Breast Cancer

**DOI:** 10.3389/fonc.2020.01683

**Published:** 2020-09-16

**Authors:** Baptiste Guey, Mélanie Bodnar-Wachtel, Annabelle Drouillard, Anaïs Eberhardt, Manon Pratviel, Nadège Goutagny, Nathalie Bendriss-Vermare, Isabelle Puisieux, Christophe Caux, Thierry Walzer, Virginie Petrilli

**Affiliations:** ^1^Centre de Recherche en Cancérologie de Lyon, INSERM U1052, CNRS UMR5286, Université de Lyon, Université Lyon 1, Centre Léon Bérard, Lyon, France; ^2^Centre International de Recherche en Infectiologie, INSERM U1111 – CNRS UMR5308, Université de Lyon, ENS de Lyon, Université Lyon 1, Lyon, France

**Keywords:** inflammasome, breast cancer, NK cells, inflammation, Caspase-1, ASC

## Abstract

Inflammasomes are molecular complexes that trigger an inflammatory response upon detection of pathogens or danger signals. Recent studies suggest that they are also involved in cancer progression. However, their roles during tumorigenesis remain poorly understood and controversial. Here, we investigated whether inflammasome activation supports mammary tumor growth. Using mouse models of invasive breast cancer, our results demonstrate that the absence of a functional inflammasome impairs tumor growth. Importantly, tumors implanted into inflammasome-deficient mice recruited significantly less neutrophils and more natural killer (NK) cells, and these latter cells displayed a more active phenotype. Interestingly, NK cell depletion abolished the anti-tumoral effect observed in inflammasome-deficient mice, although inflammasome-regulated cytokine neutralization had no effect. Thus, our work identifies a novel role for the inflammasome in supporting mammary tumor growth by attenuating NK cell recruitment and activity. These results suggest that inflammasome inhibition could be a putative target for treating invasive breast cancers.

## Introduction

Breast cancers are often infiltrated with immune cells that have pro- or anti-tumoral functions (1, 2). The composition of the immune infiltrate and the level of infiltration have been correlated with patient prognosis in several types of cancers (3–7). Chronic inflammation taking place within tumors can promote tumor progression by stimulating angiogenesis or inhibiting anti-tumoral immunity (8, 9).

Within injured tissues, innate immune cells sense pathogen- or danger-associated molecular patterns (PAMPs, DAMPs) using germline-encoded pattern recognition receptors (PRRs) that drive inflammation to restore homeostasis. Among these PRRs, specific NOD-like receptors (NLRs, such as NLRP3) and hematopoietic interferon-inducible nuclear antigens with 200 amino acid repeats (HIN200) protein families, operate by forming multiprotein complexes named inflammasomes. Besides PRRs, inflammasomes are composed of the adaptor protein associated speck-like containing a CARD protein (ASC) and of the cysteine protease caspase-1 (10). Once activated within the inflammasome complex, caspase-1 cleaves and activates two major pro-inflammatory cytokines namely the pro-IL-1β and the pro-IL-18, and drives an inflammatory cell death known as pyroptosis through gasdermin D cleavage (11, 12).

In the context of cancer, the role of the inflammasome is complex as it can both promote anti- and pro-tumoral responses. For instance, *Nlrp3-, Nlrc4*-, and *Caspase-1-*deficient mice are more sensitive to colorectal cancer induced by DSS-AOM treatment, suggesting an anti-tumoral role for the inflammasome in the gut (13–15). This protective role is mediated by the production of IL-18, which is involved in maintaining the intestinal epithelial barrier integrity. Conversely, IL-18 is a critical driver of immune suppression in a model of multiple myeloma, in which it fuels the development of myeloid-derived suppressor cells (MDSCs) (16). Moreover, the NLRP3 inflammasome was shown to promote tumor growth in models of carcinogen-induced sarcoma and skin papilloma through the release of IL-1β (17, 18). Thus, inflammasome activation may dampen or promote anti-tumor responses depending on the tumor type, the stage of tumorigenesis and the model studied. This intricacy is reinforced by the wide range of expression of some inflammasome components in immune and non-hematopoietic cells. For instance, in a carcinogen-induced skin cancer model, ASC depletion in keratinocytes facilitates tumor development, whereas its loss in myeloid cells impairs it (19).

With respect to breast cancer, the presence of IL-1β within the tumor microenvironment is frequently associated with poor prognosis, suggesting a pro-tumoral role for this cytokine (20–24). For instance, in the MMTV-Neu^*V*664*E*^ BALB/c model, the invasive conversion of the mammary tumors was associated with an upregulation of the IL-1β transcriptional signature (25). In the 4T1 murine model, which is used as a preclinical model for invasive breast cancer, IL-1β promotes tumor growth and the capacity of cells to metastasize (26, 27). Yet, the role of inflammasomes is not limited to IL-1β production and the overall impact of this pathway in the anti-breast cancer response remains unclear. We thus tested whether the inflammasome supports invasive breast cancer development *in vivo* by using mice deficient in major inflammasome components.

## Materials and Methods

### Mouse Tumor Cell Lines

4T1 and YAC-1 cells were cultured in RPMI medium supplemented with 10% (v/v) heat-inactivated FBS (Life technologies), 1% (v/v) penicillin/streptomycin, 1% (v/v) L-glutamine, and 25 μM 2-mercaptoethanol (only 4T1 cells) at 37°C in a 5% CO_2_ incubator. 4T1 cells were proven to be mycoplasma-free (MycoAlert Mycoplasma detection kit, Lonza) before each injection and experiment. Cells were also proven to be free of mouse infectious agents by Taqman® PCR testing of mouse essential panel (Charles River).

### Mice

*Nlrp3* knockout (KO) mice were obtained from J. Tschopp (28), *Asc* KO mice from V. M. Dixit (29), and *Caspase-1/Caspase-11* KO mice referred as *Caspase-1* KO in the text from R. A. Flavell (30). MMTV-Neu^*V*664*E*^ in the BALB/c from F Cavallo (31). The three transgenic KO strains were backcrossed with a BALB/c/Ola (Harlan strain) background for at least nine generations. WT animals were littermates of the *Caspase-1/Caspase-11* knockout, *Asc* knockout, or *Nlrp3* knockout colonies or imported from Harlan and maintained in the same cages as KO animals. Animals were housed in individually ventilated cages under specific pathogen-free conditions, fed with Harlan Teklad food pellets and studies were conducted in accordance with the regulations for animals used for scientific purposes governed by the European Directive 2010/63/EU. Protocols were validated by the local Animal Ethic Evaluation Committee (CECCAPP: C2EA-15, Comité d'Evaluation Commun au PBES, à AniCan, au laboratoire P4, à l'animalerie de transit de l'ENS, à l'animalerie de l'IGFL, au PRECI, à l'animalerie du Cours Albert Thomas, au CARRTEL INRA Thonon-les-Bains et à l'animalerie de transit de l'IBCP, CLB-2013-019, CLB-2015-015) and authorized by the French Ministry of Education and Research.

### Bone Marrow Mouse Chimera

Five-week-old mice received antibiotics 2 days prior to being exposed to 6 Gy g-irradiations. The day of irradiation, bone marrow (BM) was flushed with 5 mL of RPMI from the hind legs of mice and CD3^+^ cells were depleted using the CD3 MicroBead Kit (Miltenyi biotec). 10^6^ BM cells in PBS supplemented with 0.1% penicillin/streptomycin were re-injected intravenously (I.V.). Recipient animals recovered for 4 weeks before tumor injection.

### Tumor Growth Assays

Only virgin female BALB/c/Ola mice aged 7 to 10 weeks were used for *in vivo* experiments. 20,000 4T1 tumor cells in 100 μL of sterile PBS were injected orthotopically into the 4^th^ mammary fat pad. Primary tumor growth was monitored with a digital caliper measurement and expressed as a tumor volume (ellipsoidal formula, π/6 × length × width^2^). Mice were sacrificed when tumor size reached 1,200 mm^3^.

MMTV-Neu^*V*664*E*^ mice were monitored over time for tumor appearance through palpation (~100 mm^3^).

### Cell Suspensions From Spleens or Tumors

Spleens isolated from mice were crushed and filtered through a 40-μm filter and resuspended in FACS buffer (PBS supplemented with 5% (vol/vol) FBS, 2 mM EDTA). Red blood cells were lysed in 5 mL of erythrocyte lysis buffer (155 mM NH4Cl, 12 mM NaHCO3, 0.1 mM EDTA) for 5 min. After a PBS wash, cells were then resuspended in FACS buffer.

Seven or 14 days post-injection of 4T1 cells, tumors isolated from mice were cut into small pieces and incubated with 5 mL of DMEM supplemented with DNase 0.02 mg/mL (Sigma D4513)—Collagenase 1 mg/mL (Sigma C2674) for 30 min at 37°C. Digested tumors were then filtered through a 40-μm filter and re-suspended in FACS buffer and filtered again twice. Red blood cells were lysed in 5 mL of erythrocyte lysis buffer for 5 min. After a PBS wash, cells were then resuspended in FACS buffer.

### Flow Cytometry

Cell suspensions from spleens or from tumors were washed in FACS buffer (PBS supplemented with 2 mM EDTA and 5% (vol/vol) FBS) and incubated for 5 min with purified anti-mouse CD16/32 FcBlocks (93; Biolegend). Cells were stained with fluorochrome-conjugated antibodies (Supplementary Table 1) at 4°C for 20 min LIVE/DEAD^TM^ Fixable Aqua Dead Cell Stain Kit, 405 nm excitation (Invitrogen L34965) was used to gate on live cells. For cell surface staining, cells were washed twice in FACS buffer, fixed in PBS 2% paraformaldehyde and stored in FACS buffer before analysis. For intracellular staining, cells were fixed/permeabilized with the Cytofix/Cytoperm kit (554714, BD Bioscience) for 20 min on ice. Cells were washed with PermWash buffer and stained with fluorochrome-conjugated antibodies diluted (Supplementary Table 1) in PermWash buffer for 30 min on ice. Cells were washed again in PermWash buffer and kept at 4°C before analysis. Data were collected on a LSR II Fortessa (BD Bioscience) and analyzed using the FlowJo software.

### Luminex Assay

Tumors were prepared as described above. Digested tumors were centrifuged and supernatants were used for luminex assay according to manufacturer protocol (mouse pre-mixed multianalyte test reference LXAMSM-18 R&Dsystems).

### NK Cell Depletion

Mice were injected I.V. (in the retro-orbital sinus) with 50 μL of the Ultra-LEAF^TM^ Purified anti-Asialo-GM1 (clone: Poly21460, Biolegend) antibody 1 day prior to tumor cell injection. In order to maintain NK cell depletion during tumor growth, mice were injected I.V. every 10 days.

### NK Cell Activation

3.10^6^ splenic lymphocytes or tumor cell suspensions were prepared in complete medium (RPMI + glutamax, 10% SVF, 1% penicillin/streptomycin, 10 mM HEPES, 1 mM sodium-pyruvate, 50 μM 2-mercaptoethanol) and incubated for 4 h with cytokines [recombinant mouse IL-12 (Peprotech, 200-12) (final concentration: 100 ng/mL) and recombinant mouse IL-18 (R&D, B004-5) (final concentration: 20 ng/mL)] or on antibody-coated plates [anti-NKp46 (29A1; BD Biosciences), anti-Ly49D (4E5; BD Biosciences), anti-NKG2D (CX5; BD Biosciences), and GolgiStop (BD Biosciences) in the presence of anti-CD107a (2B6; BD Biosciences)] or co-cultured with Yac-1 or 4T1 cells (1:1 ratio).

### *In vivo* Cytokine Depletion

Mice were injected intraperitoneally (I.P.) with 2.5 mg/kg of body weight (B.W.) of anti-IL-1β antibody (Clone B122, Biolegend, 503504), or 0.25 mg/kg of B.W. IL-18 binding protein (IL-18BPd-FC) (R&D systems, 122-BP), or both, or control IgG 1 day prior to the tumor cell injection and every 3 days after that. Anakinra (Kineret®) was administered I.P. (20 mg/kg of B.W.) prior to tumor cell inoculation and every 2 days after that. The second anti-IL-1b (AF-401-NA; R&D Systems) or control isotype was injected i.p. at a dose of 10 μg per mouse twice a week as described in (32).

### Statistical Analysis

Statistical analysis of each experiment was conducted using the GraphPad Prism software. One-way or two-way ANOVA were used followed by Bonferroni's *Post-test* to compare tumor progression and immune cell infiltration.

## Results

### The Absence of a Functional Inflammasome Impairs Mammary Tumor Growth in Mice

**Figure 1:**
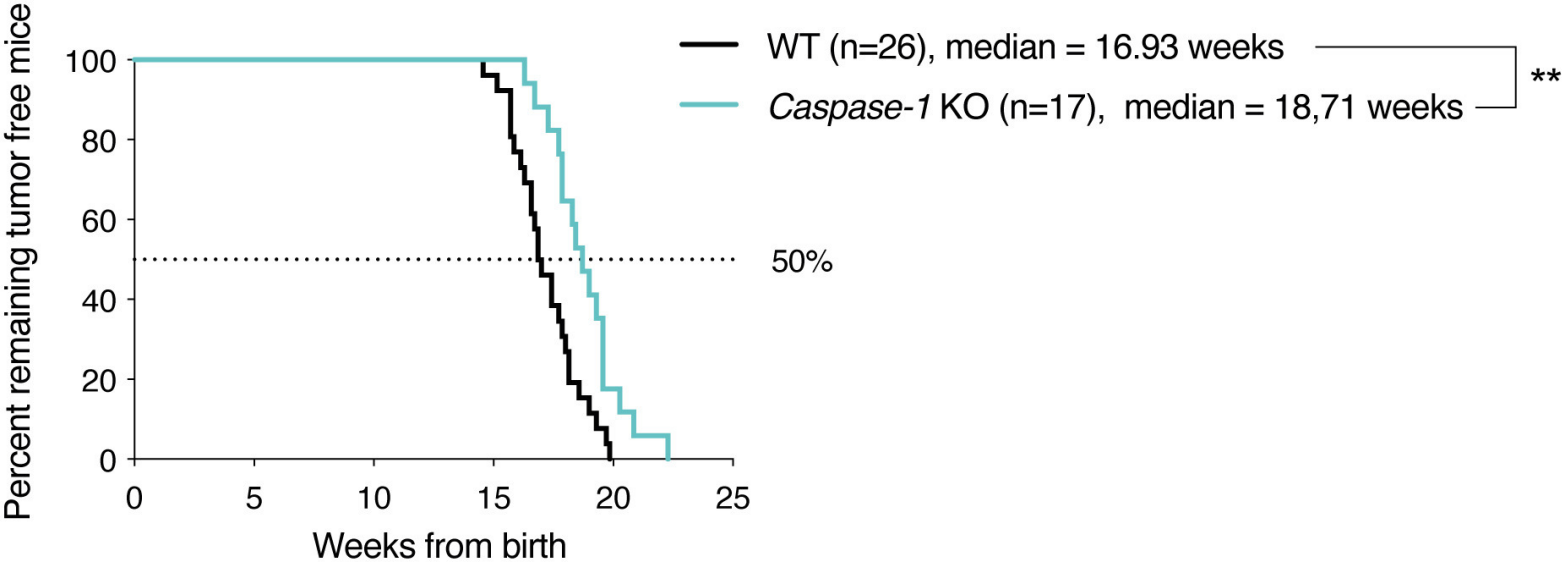
Caspase-1 deficiency delays tumor appearance in MMTV-Neu^*V*664*E*^ BALB/c mouse model. Kaplan–Meier curves depicting tumor growth latency of MMTV-Neu^V664E^ mice, defined as time from birth until appearance of the first palpable tumor. **P < 0.01 (Comparison of survival curves: Gehan–Breslow–Wilcoxon test).

**Figure 2:**
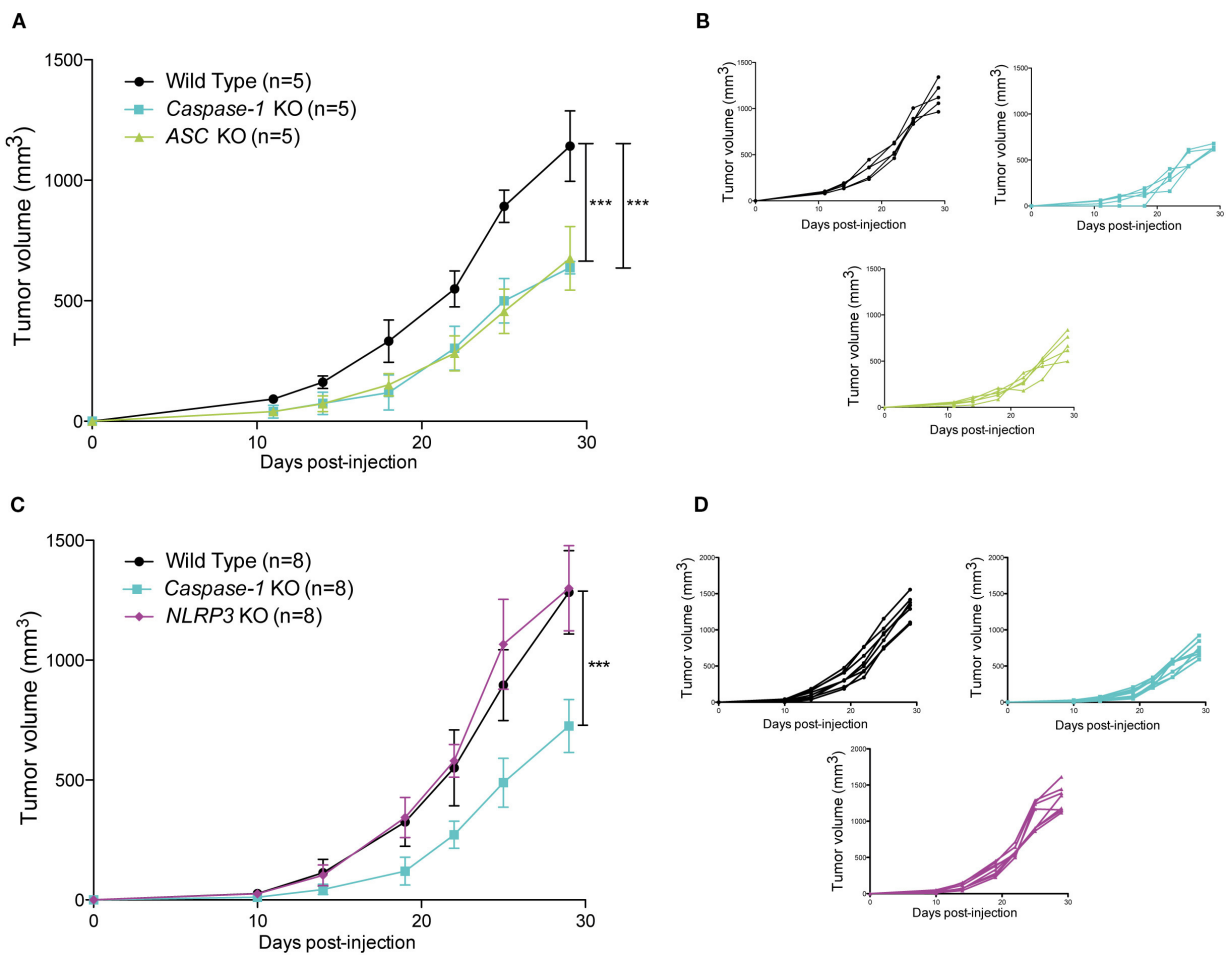
Loss of caspase-1 and ASC expression reduces 4T1 tumor growth in BALB/c mice. **(A)** WT, *Caspase-1* KO, and *Asc* KO mice (*n* = 5) were injected orthotopically with 4T1 mammary tumor cells. Tumor growth was measured over 30 days. **(B)** Individual growth curves depicted in A. **(C)** WT, *Caspase-1* KO, and *Nlrp3* KO mice (*n* = 8) were injected orthotopically with 4T1 mammary tumor cells. Tumor growth was measured over 30 days. **(D)** Individual growth curves depicted in C. Data represent mean ± SD ***P < 0.001 (Two-way ANOVA analysis; n.s, non-significant).

To assess the impact of the inflammasome on mammary cancer progression *in vivo*, MMTV-Neu^*V*664*E*^ mice were bred with *Caspase-1* knock-out (KO) mice, the main inflammasome effector, and the number of tumor-free mice was monitored over time. As shown in the Figure 1, the absence of caspase-1 significantly delayed tumor onset in mice suggesting a pro-tumoral role for the inflammasome. The difference in the age of onset was however, modest and could be due to the fact that caspase-1 is well-expressed in different tissues including mammary cells and could display opposite functions as described above for ASC (19). We thus decided to use syngeneic 4T1 carcinoma cells to explore the effect of the presence of the inflammasome within the tumor microenvironment on tumor progression. The cells were injected into the mammary fat pad of WT, *Caspase-1* KO and *Asc* KO BALB/c mice, respectively, and tumor growth was monitored over time. While inoculation of female WT BALB/c mice with cancer cells gave rise to large tumors within 30 days, the absence of caspase-1 or ASC resulted in significantly smaller tumors (Figures 2A,B), suggesting that inflammasomes likely support mammary tumor growth. To ascertain whether NLRP3 was also involved, we compared 4T1 cell growth in *Nlrp3*-deficient and *Nlrp3*-sufficient WT mice. Indeed, NLRP3 appeared to be a good candidate as this receptor is well-described to sense DAMPs, such as ATP or uric acid released by necrotic cells, and since necrosis of tumor cells is frequent during cancer progression (33, 34). As shown in Figures 2C,D, tumor sizes were similar between WT and *Nlrp3* KO mice, indicating that NLRP3 does not support mammary tumor growth *in vivo*, unlike caspase-1 and ASC.

### Inflammasome Expression in the Hematopoietic Compartment Supports Tumor Growth

**Figure 3:**
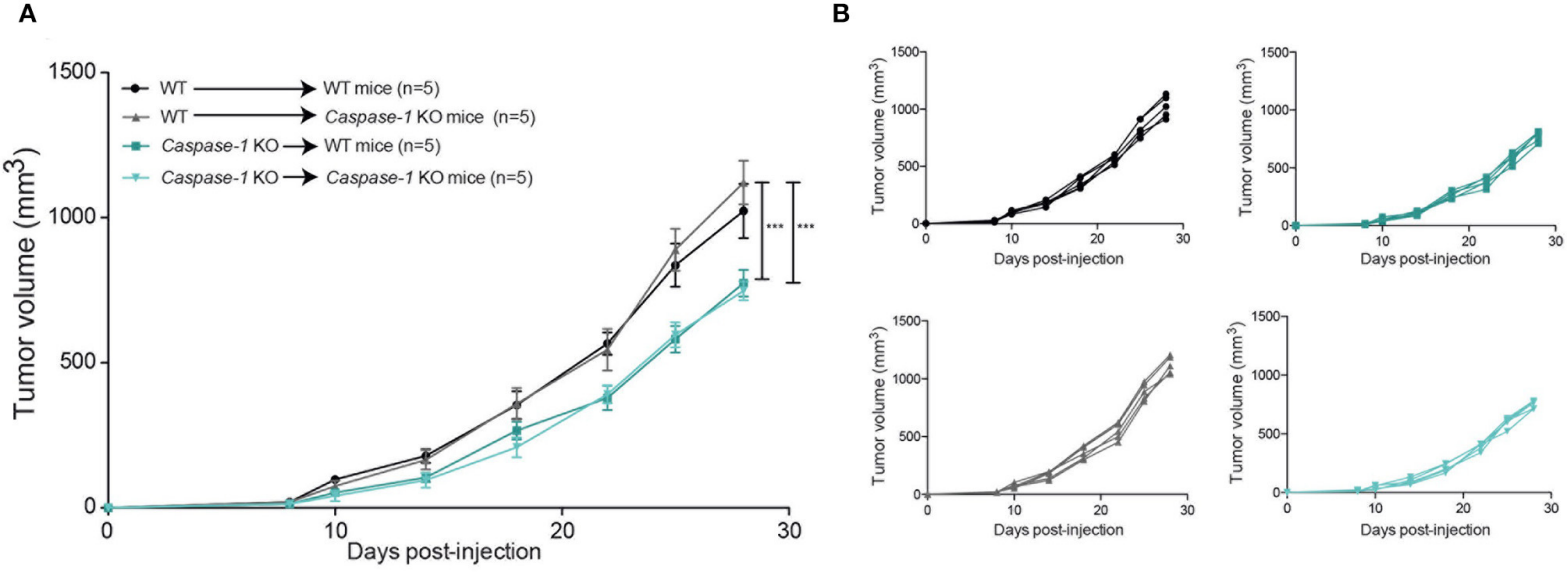
The presence of the inflammasome in the hematopoietic compartment promotes mammary tumor growth. Irradiated mice of indicated genotypes were grafted with bone marrow stem cells from WT or *Caspase-1* KO mice and were injected with 4T1 mammary tumor cells 28 days later. **(A)** Tumor growth was measured over 30 days. **(B)** Individual growth curves depicted in A. Data represent mean ± SD ***P < 0.001 (Two-way ANOVA analysis).

As previously mentioned, caspase-1 is the main catalytic subunit of the inflammasome. Its expression is not restricted to immune cells as it is also expressed by many non-hematopoietic cell types such as epithelial cells or adipocytes (35, 36). To evaluate the role of inflammasome components in tumor growth in immune vs. non-immune cells, we first generated a series of bone marrow chimeric mice to obtain different combinations of caspase-1 expression in immune and non-immune cells as indicated in Figure 3A. Thirty days post reconstitution, mice were inoculated with 4T1 cells and tumor growth was monitored. The growth rate of 4T1 cells was determined by the expression of caspase-1 in immune cells, and independent of its expression in non-immune cells, as illustrated in Figures 3A,B in recipient mice with different genotypes. Thus, the absence of caspase-1 in the hematopoietic cell lineage is responsible for the delay in tumor growth, suggesting that caspase-1-expressing immune cells support cancer progression.

### The Inflammasome Impairs NK Cell Recruitment to the Tumor

Activation of the inflammasome has been shown to modulate the composition of the tumor immune infiltrate (37). To address this finding in our model, we analyzed the tumor immune infiltrate in the mammary gland at days 7 and 14 post-injection in WT, *Asc-*, and *Caspase-1*-deficient mice. Different myeloid and lymphoid cell subtypes were studied by flow cytometry.

**Figure 4:**
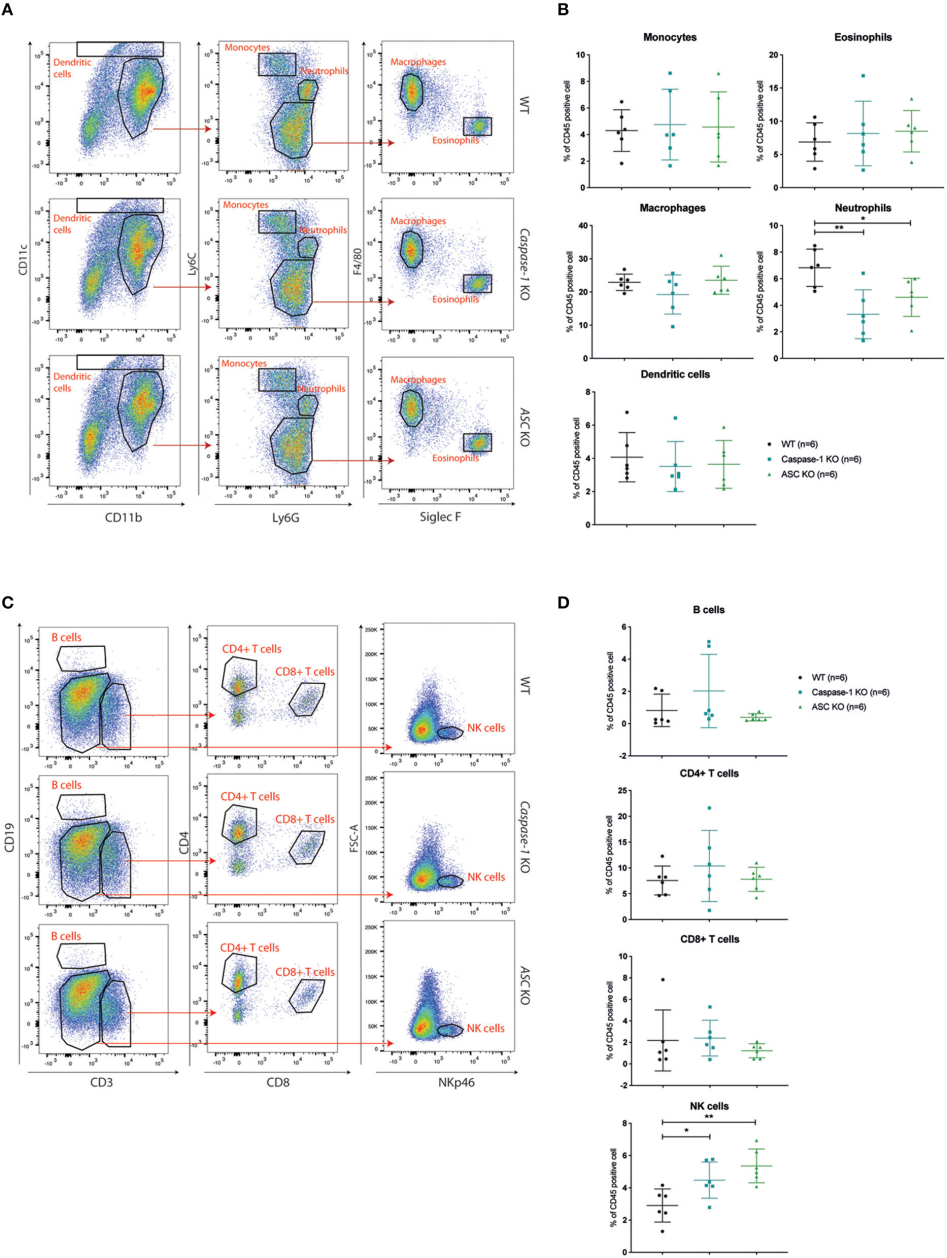
Caspase-1 or ASC deficiency improves NK cell recruitment 14 days post-injection. **(A)** Flow cytometry analysis of myeloid cell populations in 4T1 tumors at day 14 post-injection from WT (*n* = 6), *Caspase-1* KO (*n* = 6), and *Asc* KO (*n* = 6) mice. The displayed dotplots were obtained by gating live and CD45+ cells. **(B)** Quantification and analysis of myeloid cell populations in the different mouse genotypes. **(C)** Flow cytometry analysis of lymphoid cell populations in 4T1 tumors from the same mice as in A. The displayed dotplots were gated from live and CD45+ cells. **(D)** Quantification and analysis of lymphoid cell populations in the different mouse genotypes. Data represent mean ± SD *P < 0.05; **P < 0.01 (One-way ANOVA test followed by Bonferroni's Multiple Comparison Test).

With respect to the myeloid compartment, our analysis of the CD45+ infiltrate showed that the frequency of neutrophils (defined as Ly6C^int^-Ly6G^high^) was significantly decreased at both time points in *Caspase-1*- or *Asc-* deficient mice compared to control mice, while the abundance of monocytes, macrophages, dendritic cells (DC) and eosinophils was similar in all mouse groups (Figures 4A,B and Supplementary Figures 1A,C).

Regarding lymphoid cells, no significant difference in CD4^+^ or CD8^+^ T lymphocyte recruitment was observed between WT and inflammasome-deficient mice at day 7 and day 14 post-injection and very few B cells had infiltrated the tumors (Figures 4C,D and Supplementary Figure 1D). However, the frequency of infiltrating NK cells (NKp46^+^ cells) was significantly higher in tumors implanted in *Caspase-1* and *Asc* KO mice compared with WT mice at day 7 and day 14 (Figures 4C,D and Supplementary Figure 1B). Differences in tumor infiltrates between the groups of mice were not due to pre-existing differences in these mice, as the immune composition of the spleen of tumor-bearing mice was similar in WT, *Asc*, and *Caspase-1* KO mice at day 14 (Supplementary Figures 1E,F).

### Inhibition of Inflammasome-Regulated Cytokine Production Does Not Affect the Rate of Tumor Growth

**Figure 5:**
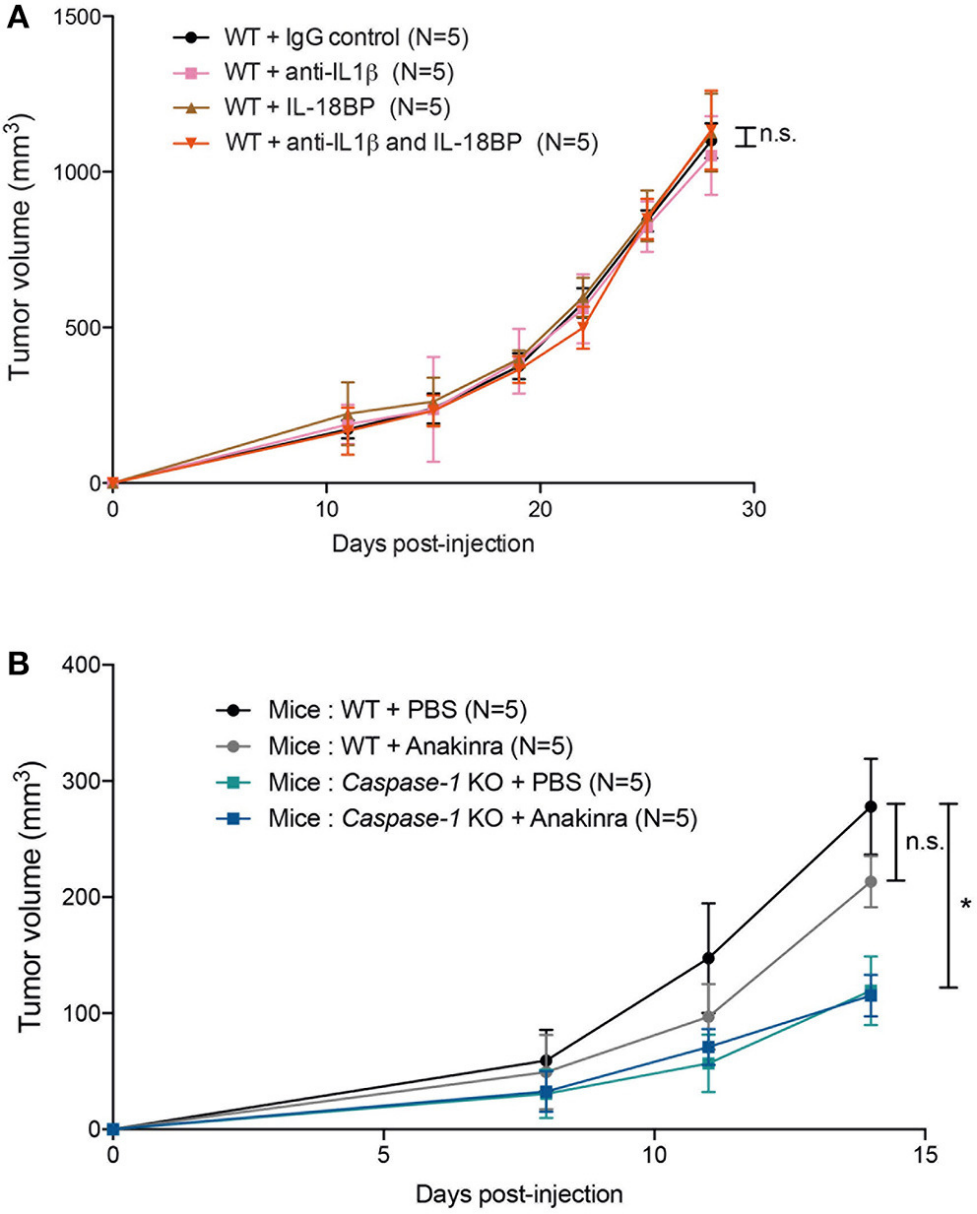
IL-1β, IL-18 or IL-1R inhibition does not affect 4T1 tumor growth *in vivo*. **(A)** WT (*n* = 5) were injected with IgG control, anti-IL1β antibody, IL-18BP, or both anti-IL1β and IL-18BP the day before tumor inoculation and then every 3 days. Treated mice were orthotopically injected with 4T1 mammary tumor cells. Tumor growth was measured over 28 days. **(B)** WT, *caspase-1* KO were injected with PBS or Anakinra (IL-1 receptor inhibitor) the day before tumor inoculation and then every 2 days. Treated mice were orthotopically injected with 4T1 mammary tumor cells. Tumor growth was measured over 14 days. Data represent mean ± SD *P < 0.05 (Two-way ANOVA analysis; n.s, non-significant).

The inflammasome controls the production of IL-1β and IL-18 and both cytokines are involved in tumor development or control. We wondered whether blocking IL-1β and IL-18 would impact the ability of 4T1 cells to grow in WT mice. Surprisingly, treatment with either the anti-IL-1β antibodies or the IL-18 binding protein (BP) or both did not affect the rate of tumor growth (Figure 5A, Supplementary Figure 2). Similarly, Anakinra (recombinant IL-1Ra) injection did not delay tumor growth in WT mice, suggesting that inflammasome-regulated cytokines are not essential for controlling 4T1 cell growth *in vivo* (Figure 5B) (24, 32, 38). Finally, cytokine measurement using multiplex technology of tumor supernatants at day 14 showed no difference in the amount of IL-1β, IL-33, CCL3/MIP1α, KC or β-FGF, between WT and *Caspase-1* KO, while CCL5/RANTES was significantly increased in *Caspase-1* KO (Supplementary Figure 3).

### Caspase-1 Deficiency Improves NK Cell Anti-tumor Activity

**Figure 6:**
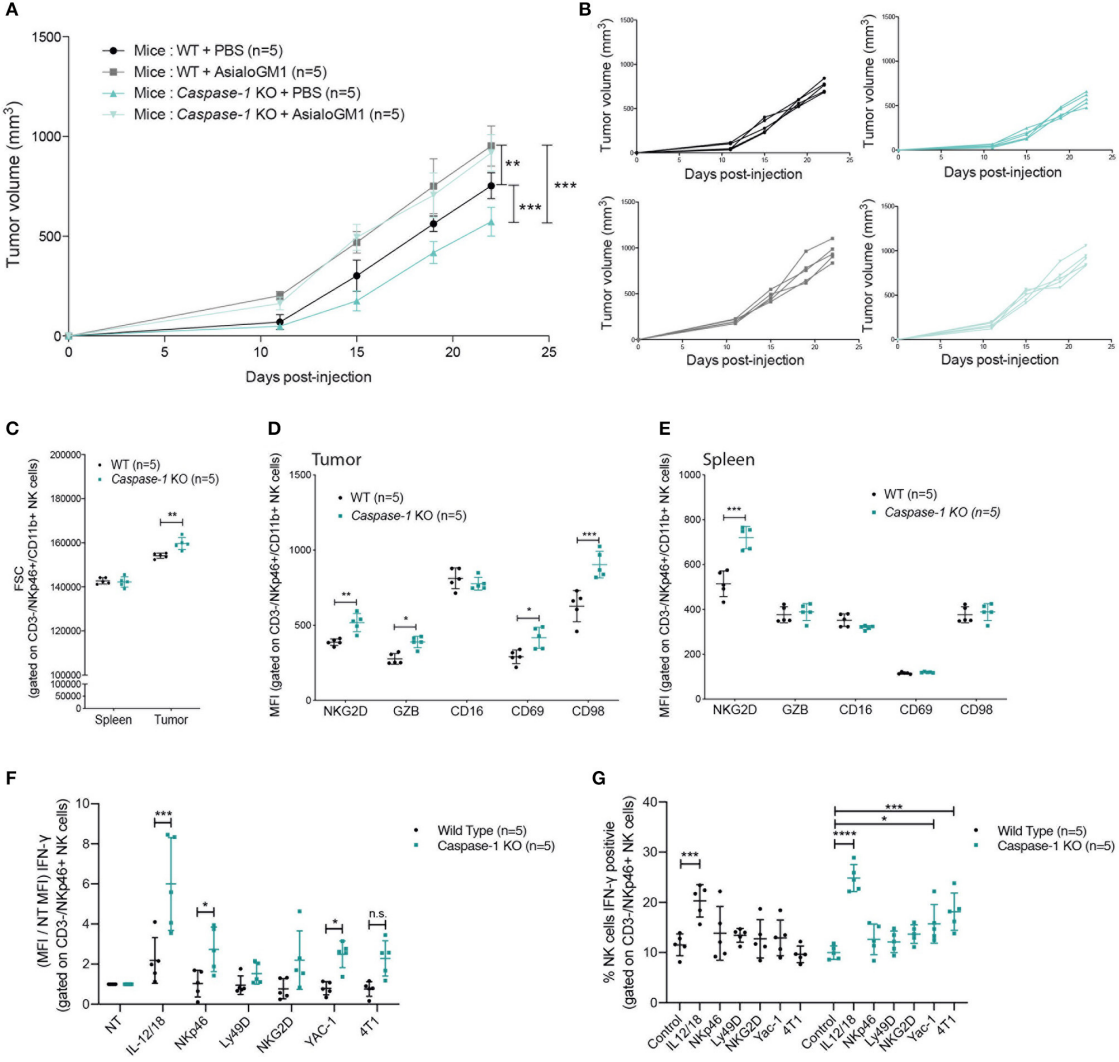
NK cells control tumor growth and are more active in *caspase-1* KO mice. **(A)** Tumor growth of 4T1 cells was measured in NK-depleted (anti-asialo GM1) or PBS-treated WT and *caspase-1* KO mice (*n* = 5). Each value represents mean ± SD **P < 0.01; ***P < 0.001 (Two-way ANOVA analysis). **(B)** Individual growth curves depicted in **(A)**. **(C–E)** Flow cytometry analysis of NK cell size in tumors and spleens **(C)** and activation markers in 4T1 tumors **(D)** and in spleen **(E)** from WT (*n* = 5) and *Caspase-1* KO (*n* = 5) mice at day 7 post-injection. **(F)** Cell suspensions from digested tumors of the indicated mouse genotype were cultured in the presence of cytokines (IL-12/IL-18), antibodies (NKp46, Ly49D, NKG2D) or tumor cells (YAC-1, 4T1) and NK cell IFN-γ production **(F)** or IFN-γpositive cells **(G)** were measured by flow cytometry. Data from **(C–G)** represent mean ± SD *P < 0.05; **P < 0.01; ***P < 0.001; ****P < 0.0001 (Two-way ANOVA test followed by Bonferroni's Post-test; ns, non-significant).

Since NK cells, which are important anti-tumor effectors (39, 40), were preferentially recruited into the tumors of inflammasome-deficient mice, and since increased levels of CCL5 within the tumor microenvironment were detected, we tested whether NK cells were responsible for the reduction in tumor growth. *Caspase-1* KO and WT mice were depleted of NK cells by I.V. injection of the anti-Asialo GM1 antibody before being inoculated with 4T1 cells. Upon NK cell depletion, tumors grew at the same rate in both groups of mice, demonstrating a major role for NK cells in mammary tumor growth control (Figures 6A,B).

We then asked whether caspase-1 expression within the immune compartment could affect NK cell phenotype and activation. As shown in Figure 6C, NK cells infiltrating the mammary tumors of *Caspase-1* KO were larger (according to FSC parameters) than those infiltrating WT mice, a feature which is commonly associated with a stronger activation status (41). Furthermore, NK cells present in the tumor of *Caspase-1*-deficient hosts expressed higher levels of NK cell activation markers NKG2D, Granzyme B (GZB), CD69 and CD98 (Figure 6D) (42). With the exception of NKG2D, few differences were detected between splenic NK cells from WT and *Caspase-1*-deficient tumor-bearing mice, indicating that the tumor microenvironment directly impacts NK cell phenotype (Figure 6E).

We then investigated the ability of tumor infiltrating NK cells to be activated when re-stimulated *in vitro*. Tumor cell suspensions from WT or *Caspase-1* KO mice were incubated with a combination of IL-12+IL-18 cytokines, or with crosslinking antibodies against activating receptors (NKp46, Ly49D, NKG2D) or with different tumor cell lines (4T1, and YAC1 cells, a classical NK cell target). Interestingly, NK cells from tumors growing in *Caspase-1* KO mice displayed an increased expression of IFN-γ following activation compared to those from tumors growing in WT mice, irrespective of the stimulus used (Figure 6F and Supplementary Figure 4A). In addition, in the tumor cell suspension from *Caspase-1* KO mice, significantly more NK cells were activated (positive to IFN-γ) in response to YAC-1 or 4T1 stimulation compared with WT (Figure 6G and Supplementary Figure 4B). These results suggest that the absence of caspase-1 from the tumor immune microenvironment promotes NK cell activation.

## Discussion

The role of inflammasome activation in cancer (15, 43) remains largely undefined and can be either pro-tumoral or anti-tumoral. The data presented here suggest that the inflammasome promotes the growth of invasive breast cancer in two mouse models, the MMTV-Neu and 4T1 cells. Interestingly, bone marrow chimeric mouse experiments demonstrated that caspase-1-expressing immune cells promote mammary tumor progression.

Despite the fact that necrotic cell death occurs during tumor progression releasing DAMPs, such as ATP, or uric acid, tumor growth was independent of NLRP3, suggesting either redundancy or the involvement of another PRR. Other innate immune receptors such as AIM2 or NLRC4 can form inflammasome platforms. AIM2 is a DNA sensor, which can be activated by circulating-free DNA released by dying cells (44, 45). And NLRC4 was recently shown to promote mammary tumor growth in a model of high fat diet-induced obesity via the production of IL-1β (46). Further experiments would be required using KO animals in the BALB/c background to determine their putative involvement in tumor progression of invasive breast cancer.

NK cells are an important aspect of the anti-tumor arsenal and their presence is associated with good prognosis in several types of cancers (47, 48). However, during cancer progression malignant cells develop different strategies to escape or to dampen NK cell functions (39, 49). Indeed, NK cell activity was shown to be reduced in the blood of primary and metastatic breast cancer patients (50).

With respect to the role of the inflammasome on NK cell function, former studies mostly addressed its involvement in mouse models of cancer metastasis (15). For instance, *Nlrp*3-deficient mice displayed reduced number of melanoma lung metastasis due to more active NK cells in a caspase-1-independent way, while in the context of colon metastasis to the liver, caspase-1 and NLRP3 were protective by promoting more active NK cells in the livers of WT mice compared with *Caspase-1*-deficient mice (17, 37). Here, we showed for the first time that the absence of a functional inflammasome improved NK cell recruitment and activation in the mammary tumor microenvironment. Higher levels of CCL5 were also detected in the tumor supernatant of *Caspase-1*-deficient tumors consistent with an increase in NK cells recruitment and activation (51, 52). We further showed that NK cells from *Caspase-1*-deficient mice responded better to *ex vivo* re-stimulations, and NK cell depletion in WT and *Caspase-1*-deficient mice resulted in similar tumor growth rates, demonstrating the major role of NK cells on tumor growth control. Since the phenotype of NK cells was similar in WT and in *Caspase-1* KO mouse spleens, our results suggest that the tumor microenvironment directly modulates NK cell anti-tumor response, as previously described in invasive breast cancer and non-small cell lung cancer (NSCLC) models (53, 54).

Our observations also revealed that Ly6C^int^-Ly6G^high^ neutrophils were less abundant in the tumor microenvironment of inflammasome-deficient mice. Several studies have described these tumor infiltrating cells as myeloid-derived suppressor cell (MDSC) populations, due to their ability to down-regulate the anti-cancer immune response (55, 56). For instance, MDSC recruited within the tumor bed are able to suppress NK cell cytotoxicity, IFN-γ production and NKG2D expression (57). MDSCs promote primary and metastatic 4T1 tumor progression (26), and impairing their recruitment to the tumor microenvironment limits tumor growth (22, 58). Intriguingly, Chow et al. reported in *Nlrp3* KO lungs the presence of a CD11b^+^ Gr-1^int^ population which, upon adoptive transfer into WT animals, suppressed lung metastasis of melanoma cells (17). Moreover, those cells secreted CCL5. However, we did not observe the recruitment of a similar population in the mammary immune infiltrate of *Caspase-1* or *Asc* KO mice. Thus, our data suggest that the inflammasome supports tumor growth by recruiting Ly6C^int^-Ly6G^high^ cells to the tumor bed preventing NK cell infiltration and activation.

Activation and secretion of the two pro-inflammatory cytokines IL-1β and IL-18 are mostly regulated through the inflammasome. According to previous studies, IL-1β promotes 4T1 tumor growth (26, 27). However, we did no detect any difference in intra-tumoral IL-1β concentration and the inhibition of the IL-1R pathway by IL-1Ra administration or through the immune-depletion of IL-1β *in vivo* did not impair 4T1 growth. Moreover, injecting the same anti-IL-1β used by Kaplanov et al. in WT mice did not affect 4T1 growth, although tumor volumes were globally much smaller in their study compared with ours (32). In addition, Bruchard et al. described no decrease in tumor growth in the presence of IL-1Ra (59). IL-18 inhibition, and the combination of both IL-1β and IL-18 inhibition also had no impact on tumor cell growth. The discrepancies observed these different studies could be explained by the BALB/c strain that we used or by specific in-house microbiota. Of note, using the aggressive PyMT mouse model of invasive breast carcinoma inter-crossed with the *Il-1r* KO background, Dagenais and colleagues observed an increase in tumor burden and aggressiveness, while no effect on the composition of the tumor immune microenvironment was noted, minimizing the role of the IL-1R pathway as a main modulator of breast cancer progression through the modulation of the immune composition (60). Thus, in our model, the inflammasome may support tumor growth through as yet understudied effector mechanisms. They could be pyroptosis, which induces pore formation in the plasma membrane and the release of the intracellular content, or caspase-1 mediated eicosanoid storm (12, 61). Eicosanoids, and especially prostaglandin E2 (PGE2) synthesized by cyclooxygenases, were known to suppress anti-tumor immunity by inhibiting NK cell viability and activation, and to promote cancer growth (51, 62, 63).

Altogether, our study highlights a new role for the inflammasome in promoting invasive breast cancer progression by facilitating tumor infiltration with neutrophils, while impeding the NK cell-associated anti-tumoral response independently of IL-1β and IL-18. These results suggest that inflammasome catalytic inhibition could be an interesting therapeutic approach for breast cancer.

## Data Availability Statement

The raw data supporting the conclusions of this article will be made available by the authors, without undue reservation.

## Ethics Statement

The animal study was reviewed and approved by the local Animal Ethic Evaluation Committee (CECCAPP: C2EA-15, Comité d'Evaluation Commun au PBES, à AniCan, au laboratoire P4, à l'animalerie de transit de l'ENS, à l'animalerie de l'IGFL, au PRECI, à l'animalerie du Cours Albert Thomas, au CARRTEL INRA Thonon-les-Bains et à l'animalerie de transit de l'IBCP, CLB-2013-019, CLB-2015-015) and authorized by the French Ministry of Education and Research.

## Author Contributions

BG, TW, and VP designed experiments. BG, MB-W, AD, MP, IP, AE, and VP performed research. NG, NB-V, CC, and TW contributed to agents/analytic tools. BG, TW, and VP analyzed data. BG and VP wrote the paper. All authors contributed to the article and approved the submitted version.

## Conflict of Interest

The authors declare that the research was conducted in the absence of any commercial or financial relationships that could be construed as a potential conflict of interest.

## Supplementary Material

Supplementary Figure 1 Caspase-1 and ASC deficiency alters the composition of the tumor immune infiltrate in tumors but not that of the spleen. **(A)** Flow cytometry quantification of CD45 positive cells in 4T1 tumors isolated from WT (*n* = 6), *Caspase-1* KO (*n* = 6), and *Asc* KO (*n* = 6) mice 14 days post-injection. **(B)** Correlation graph of NK cells vs. neutrophils infiltrated in 4T1 tumors isolated from WT (*n* = 6), *Caspase-1* KO (*n* = 6), and *Asc* KO (*n* = 6) mice 14 days post-injection. **(C,D)** Flow cytometry quantification and analysis of myeloid **(C)** and lymphoid **(D)** cell populations from WT, *Caspase-1* KO, and *Asc* KO mice 7 days post-injection with 4T1 mammary tumor cells. **(E,F)** Flow cytometry quantification and analysis of myeloid **(E)** and lymphoid **(F)** cell populations in spleens from WT (*n* = 4), *Caspase-1* KO (*n* = 4), and *Asc* KO (*n* = 4) mice injected with 4T1 mammary tumor cells at day 14 post 4T1 tumor cell injection. Data represent mean ± SD ^*^*P* < 0.05, ^**^*P* < 0.01 (One-way ANOVA test followed by Bonferroni's Multiple Comparison Test).

Supplementary Figure 2 IL-1β inhibition does not affect 4T1 tumor growth *in vivo*. WT mice were injected with IgG control (*n* = 7) or anti-IL1β antibody (*n* = 8) the day before tumor inoculation and then twice a week. Treated mice were orthotopically injected with 4T1 mammary tumor cells. Tumor growth was measured over 28 days.

Supplementary Figure 3 Cytokine measurements in tumor cell supernatants of WT or *caspase-1* KO mice by Luminex technology. Supernatants from tumor dilacerations of WT (*N* = 8) and *caspase-1* KO (*N* = 8) mice were analyzed by Luminex assay for CCL5 IL-1β, CCL3, IL-33, KC, and FGF-b. Data represent mean ± SD (*p* from unpaired *t*-test).

Supplementary Figure 4 NK cells control tumor growth and are more activated in *caspase-1* KO mice. **(A)** Cytometric profiles of data shown in Figure 6F. Cell suspensions from digested tumors of the indicated mouse genotype were cultured in the presence of cytokines (IL-12/IL-18), antibodies (NKp46, Ly49D, NKG2D), or tumor cells (YAC-1, 4T1) and NK cell IFN-γ production was measured by flow cytometry. **(B)** Comparison of IFN-γ-positive NK cells from tumor of WT and Caspase-1 KO mice exposed or not to 4T1 cells.

Supplementary Table 1 Inventory of fluorochrome conjugated-antibodies used for cytometry analysis.
