# Supplementary figures and images for: Inflammasome Deletion Promotes Anti-tumor NK Cell Function in an IL-1/IL-18 Independent Way in Murine Invasive Breast Cancer
Source: Front Oncol. 2020 Sep 16;10:1683. doi: 10.3389/fonc.2020.01683 (PMC7526436; doi:10.3389/fonc.2020.01683)

# Guey B. et al, Supplementary figure 1

A

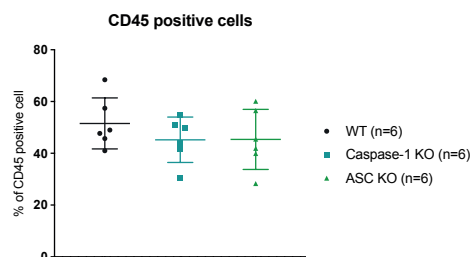

B

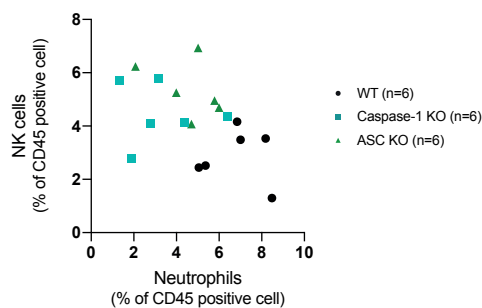

C

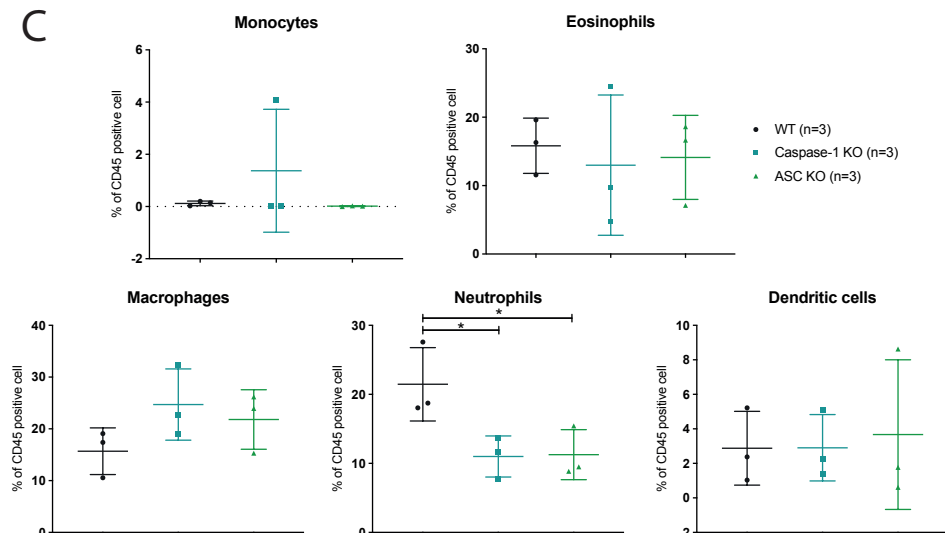

D

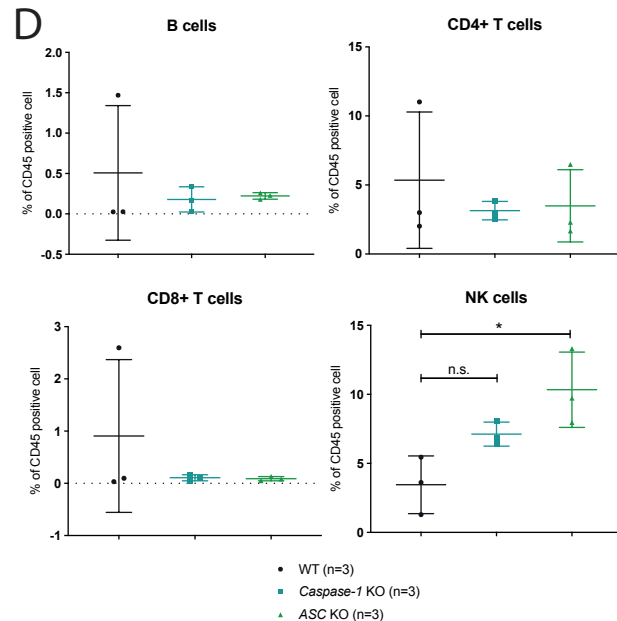

Spleen

E

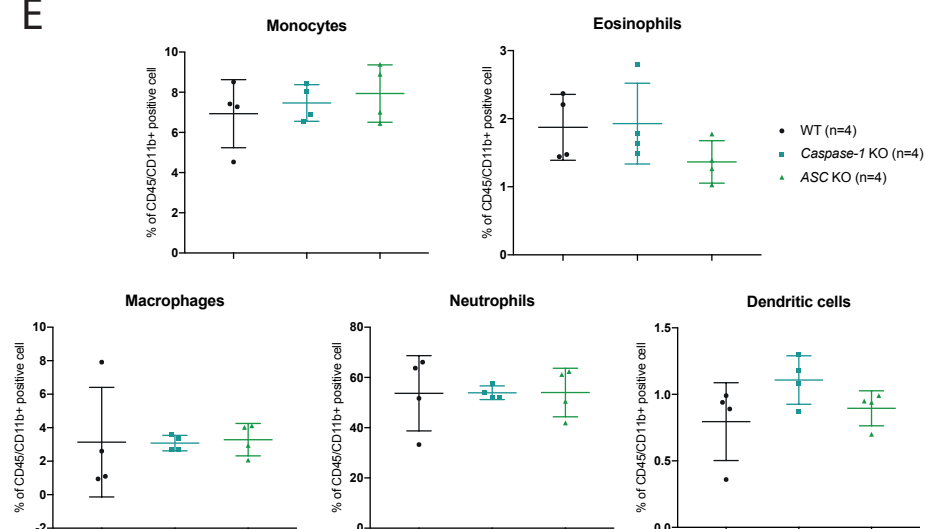

F

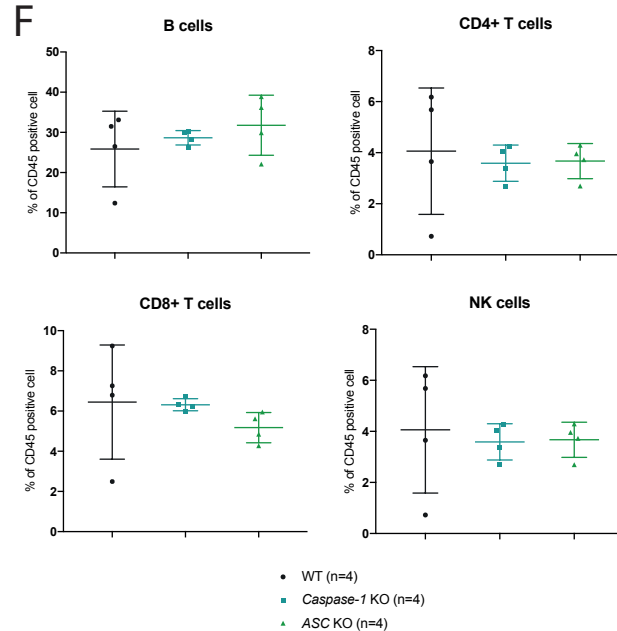

Supplement: Supplementary Figure 1 — Caspase-1 and ASC deficiency alters the composition of the tumor immune infiltrate in tumors but not that of the spleen. (A) Flow cytometry quantification of CD45 positive cells in 4T1 tumors isolated from WT (n = 6), Caspase-1 KO (n = 6), and Asc KO (n = 6) mice 14 days post-injection. (B) Correlation graph of NK cells vs. neutrophils infiltrated in 4T1 tumors isolated from WT (n = 6), Caspase-1 KO (n = 6), and Asc KO (n = 6) mice 14 days post-injection. (C,D) Flow cytometry quantification and analysis of myeloid (C) and lymphoid (D) cell populations from WT, Caspase-1 KO, and Asc KO mice 7 days post-injection with 4T1 mammary tumor cells. (E,F) Flow cytometry quantification and analysis of myeloid (E) and lymphoid (F) cell populations in spleens from WT (n = 4), Caspase-1 KO (n = 4), and Asc KO (n = 4) mice injected with 4T1 mammary tumor cells at day 14 post 4T1 tumor cell injection. Data represent mean ± SD *P < 0.05, **P < 0.01 (One-way ANOVA test followed by Bonferroni's Multiple Comparison Test). [file Image_1.pdf]

Guey B. et al, Supplementary figure 2

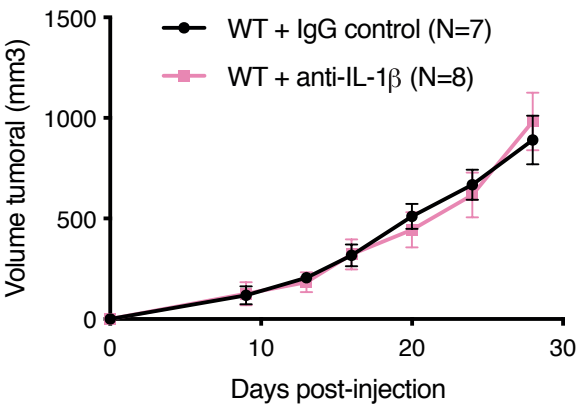

Supplement: Supplementary Figure 2 — IL-1β inhibition does not affect 4T1 tumor growth in vivo. WT mice were injected with IgG control (n = 7) or anti-IL1β antibody (n = 8) the day before tumor inoculation and then twice a week. Treated mice were orthotopically injected with 4T1 mammary tumor cells. Tumor growth was measured over 28 days. [file Image_2.pdf]

Guey B. et al, Supplementary figure 3

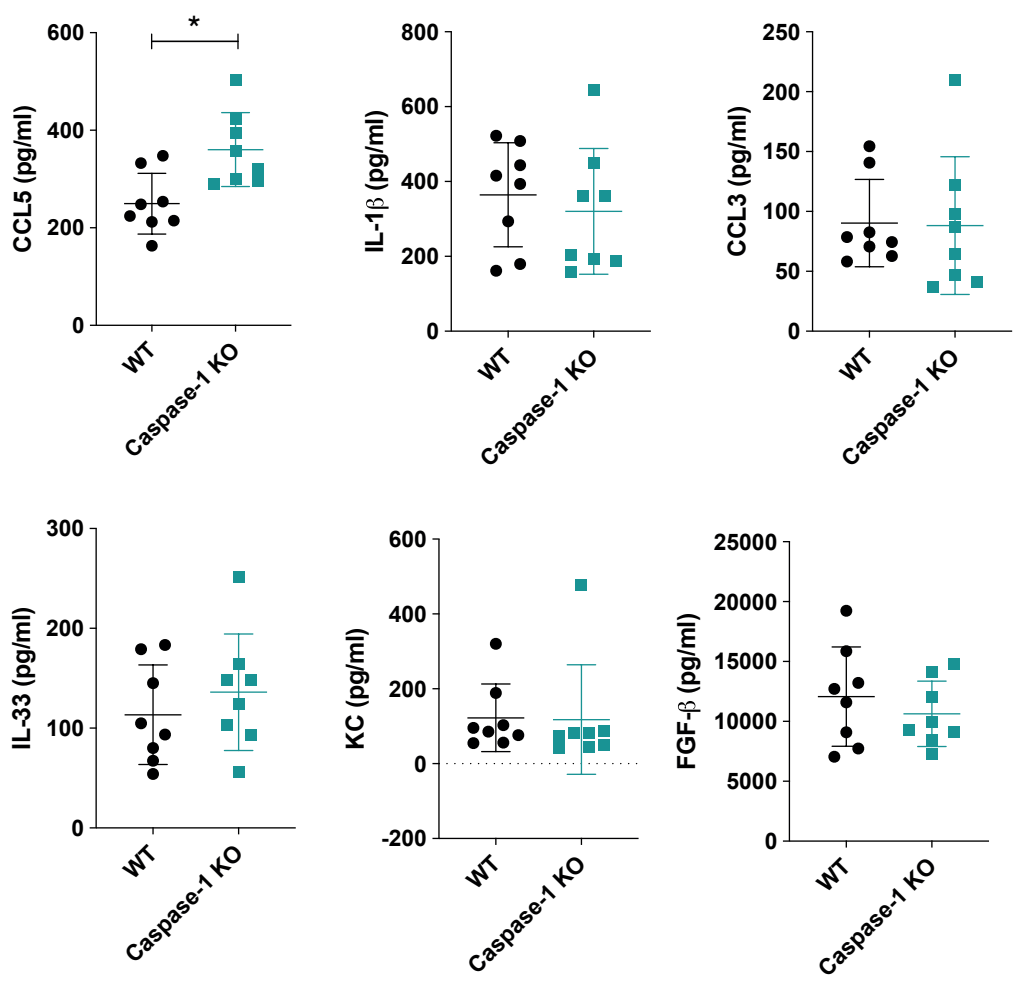

Supplement: Supplementary Figure 3 — Cytokine measurements in tumor cell supernatants of WT or caspase-1 KO mice by Luminex technology. Supernatants from tumor dilacerations of WT (N = 8) and caspase-1 KO (N = 8) mice were analyzed by Luminex assay for CCL5 IL-1β, CCL3, IL-33, KC, and FGF-b. Data represent mean ± SD (p from unpaired t-test). [file Image_3.pdf]

Guey B. et al, Supplementary figure 4

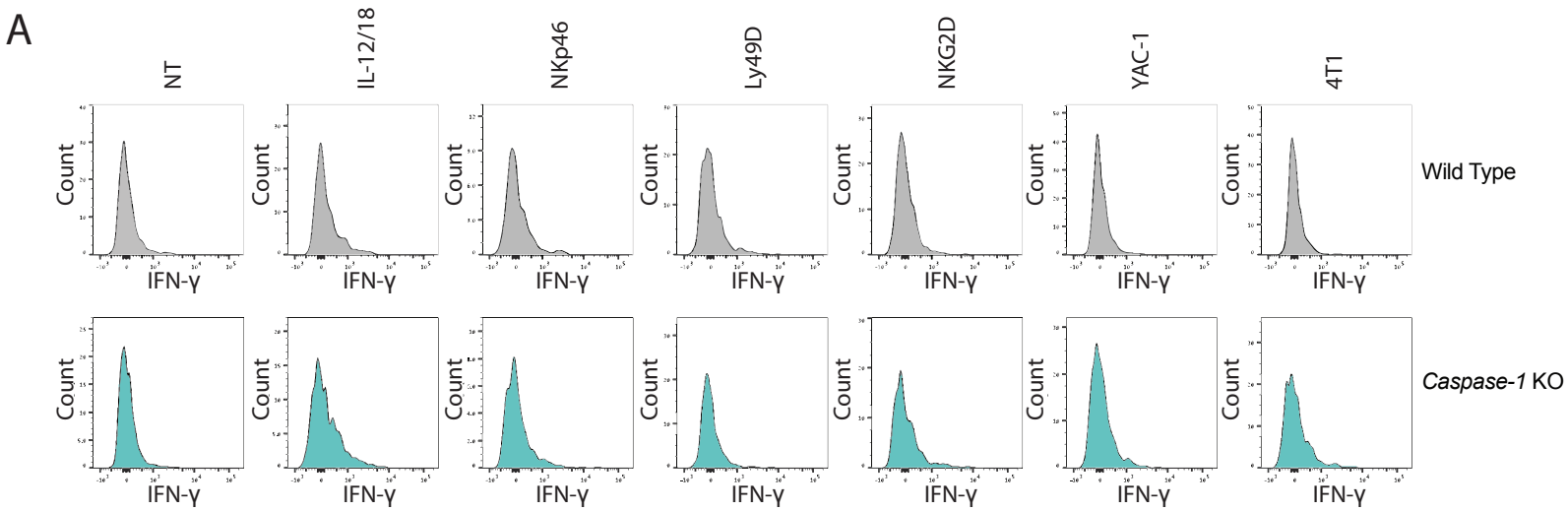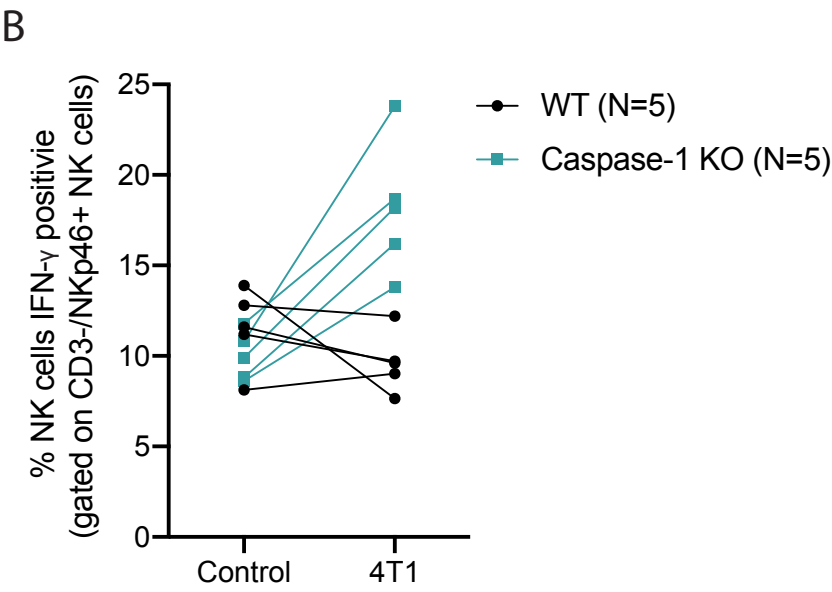

Supplement: Supplementary Figure 4 — NK cells control tumor growth and are more activated in caspase-1 KO mice. (A) Cytometric profiles of data shown in Figure 6F. Cell suspensions from digested tumors of the indicated mouse genotype were cultured in the presence of cytokines (IL-12/IL-18), antibodies (NKp46, Ly49D, NKG2D), or tumor cells (YAC-1, 4T1) and NK cell IFN-γ production was measured by flow cytometry. (B) Comparison of IFN-γ-positive NK cells from tumor of WT and Caspase-1 KO mice exposed or not to 4T1 cells. [file Image_4.pdf]
