# Supplementary material for: Inflammasome Deletion Promotes Anti-tumor NK Cell Function in an IL-1/IL-18 Independent Way in Murine Invasive Breast Cancer
Source: Front Oncol. 2020 Sep 16;10:1683. doi: 10.3389/fonc.2020.01683 (PMC7526436; doi:10.3389/fonc.2020.01683)
Supplement: Supplementary Table 1 — Inventory of fluorochrome conjugated-antibodies used for cytometry analysis. [file Table_1.pdf]

Guey B. et al, Supplementary table 1

Extracellular staining

Antigen	Clone	Conjugate	Ref/Supplier
CD11b	M1/70	PerCP-Cy5.5	101228 Biolegend
CD11c	N418	Brilliant Violet 605	117333 Biolegend
CD16	275003	APC	FAB19601A, R&D
CD19	6D5	Brilliant Violet 605	115540 Biolegend
CD3	17A2	APC/Cy7	100222 Biolegend
CD4	RM4-5	PerCP-Cy5.5	550954 BD Biosciences
CD45	30-F11	Alexa Fluor 700	B191240 Biolegend
CD69	H1.2F3	Alexa Fluor 488	104516 Biolegend
CD8	53-6.7	PE	100708 Biolegend
CD98	RL388	PE	120981 eBioscience
F480	BM8	Alexa Fluor 488	123120 Biolegend
Ly6C	AL-21	APC/Cy7	560596 BD Biosciences
Ly6G	1A8	Brilliant Violet 570	127629 Biolegend
NKG2D	CX5	PE	12-5882, eBioscience
NKp46	29A1.4	APC	137608 Biolegend
SiglecF	E50-2440	PE	552126 BD Biosciences

intracellular stanning

Antigen	Clone	Conjugate	Ref/Supplier
Granzyme B	GB12	PE	MHGB04, Invitrogen
IFN-Gamma	XMG1.2	PE	554412, BD Biosciences
